# Human neutrophils recognize group B streptococci *via* formylated peptide receptors and toll-like receptor 8

**DOI:** 10.3389/fimmu.2026.1828994

**Published:** 2026-05-04

**Authors:** Luigi Fiore, Giuseppe Valerio De Gaetano, Federica Grasso, Francesco Coppolino, Agata Famà, Mariachiara Stifano, Annamaria Petrungaro, Federica Vita, Eugenia Quartarone, Chiara Cipollina, Germana Lentini, Concetta Beninati

**Affiliations:** 1 Department of Human Pathology, University of Messina, Messina, Italy; 2 Unity of Transfusion Medicine, University Hospital Gaetano Martino, Messina, Italy; 3 Ri.MED Foundation, Palermo, Italy; 4 Scylla Biotech Srl, Messina, Italy

**Keywords:** bacterial RNA, formylated peptides, gram positive bacteria, IL-8, pattern recognition receptors, reactive oxygen species

## Abstract

**Introduction:**

Neutrophils, the most abundant leukocyte population, play a central role in host defense against *group B Streptococcus* (GBS), an encapsulated bacterium that frequently causes severe infections in humans, including sepsis and meningitis. However, the molecular pathways by which human neutrophils detect and respond to GBS remain incompletely defined.

**Methods:**

Neutrophils were isolated from the peripheral blood of healthy donors and stimulated with live and killed GBS or their nucleic acids, in the presence of specific inhibitors of immune recognition receptors. Cytokine responses were assessed by enzyme-linked immunosorbent and quantitative polymerase chain reaction assays, while reactive oxygen species were detected using fluorescence flow cytometry.

**Results:**

We initially found that neutrophils produce similar levels of several pro-inflammatory cytokines when stimulated with either live or heat-killed GBS. However, live GBS induce significantly higher levels of interleukin-8 (IL-8) and reactive oxygen species (ROS) compared to killed bacteria by a mechanism that requires activation of formyl peptide receptors 1 or 2 (FPR1 or FPR2). We identified two formylated signal peptides (f-pep8 and f-pep10) which selectively activate FPR1 and FPR2, respectively, promoting neutrophil chemotaxis and ROS generation. We also found that Toll-like receptor 8 (TLR8), but not TLR2, is required for recognition of both live and killed GBS through its ability to recognize bacterial RNA.

**Discussion:**

These findings reveal a tripartite sensing mechanism involving TLR8, FPR1 and FPR2, which together amplify neutrophil responses to live GBS. While FPR1 and FPR2 signaling are critical for the augmented production of IL-8 and ROS, they are dispensable for the induction of most other pro-inflammatory cytokines. These data may be useful for better understanding the mechanisms by which neutrophils sense the presence of GBS and for guiding the development of therapeutic strategies targeting innate immune pathways in bacterial infections.

## Introduction

*Streptococcus agalactiae*, or group B *Streptococcus* (GBS), is a common colonizer of the human gastrointestinal tract that can cause a wide range of serious infections, including sepsis, meningitis, endocarditis, soft tissue infections and arthritis in vulnerable individuals (1). The incidence of infections in adults has been steadily increasing, particularly in the elderly and in patients with underlying chronic conditions (1–4). In neonates, GBS remains a leading cause of severe diseases, including sepsis and meningoencephalitis, which can result in long-term neurological impairment (5, 6). The recognition of GBS by the innate immune system is crucial for protecting the host against these infections (7). Upon detecting GBS or other bacteria, the innate immune system produces a cascade of cytokines that recruit neutrophils, enhance phagocytosis and promote bacterial clearance (7–9). The recruitment of neutrophils to the infection site is essential for effective anti-GBS host defenses. For example, experimental neutrophil depletion in animal models leads to overwhelming septicemia and death under conditions in which control animals can prevent GBS growth and clear the infection (7). Additionally, clinical studies have shown that neonates with reduced neutrophil counts are more vulnerable to GBS-induced sepsis (8, 9). Despite the critical role of neutrophils, the specific molecular mechanisms by which they recognize and respond to GBS remain unclear.

Host immune responses are initiated when pathogen recognition receptors (PRRs), such as Toll-like receptors (TLRs), detect microbial components called pathogen-associated molecular patterns (PAMPs; 8). These evolutionarily conserved molecules, found across a broad range of microorganisms, serve as signals that alert PRR-expressing immune cells to the presence of infection. To prevent excessive reactions, the immune system calibrates its response according to the perceived severity of the threat, which is gauged using three main factors (1): the presence and concentration of PAMPs in normally sterile tissues; (2) the detection of damage-associated molecular patterns (DAMPs), which signal tissue injury; (3) the ability to distinguish between live and dead microbes, with a stronger immune response typically mounted against live pathogens (10). This live/dead discrimination ability has important consequences, as shown by the more robust protection provided by live vaccines compared with inactivated ones (11, 12). Although the underlying mechanisms are not fully understood, it has been suggested that so-called “vita-PAMPs” (*i.e.* pathogen products uniquely associated with viable microorganisms, such as labile bacterial RNA) can elicit stronger immune activation (13, 14). Specifically, mononuclear phagocytes appear to utilize Toll-like receptor 8 (TLR8) to sense live organisms by detecting their single stranded RNA, a labile molecular species less abundant in dead microbes (10). Indeed, TLR8 has recently emerged as a central bacterial sensor, due to its capacity to detect pathogen RNA (15, 16) and drive robust T-helper 1 (Th1) responses through the induction of a distinctive cytokine profile (17). Moreover, recent data have further clarified the molecular mechanism underlying TLR8 activation, by showing that this receptor becomes engaged by uridine and uridine-terminated breakdown products generated by various RNases upon processing of microbial RNA (18, 19). Formyl peptide receptors (FPRs) are a class of PRRs that play a pivotal role in guiding neutrophils to infection sites by detecting formylated peptides and other bacterial products functioning as PAMPs. FPRs also stimulate the production of reactive oxygen species (ROS), which have potent antimicrobial effects, and attract neutrophils to infection sites (20). The number of FPR types varies in different species, with 3 FPR genes identified in humans: FPR1, FPR2, and FPR3. Although FPR1 and FPR2 are closely related, sharing 69% sequence identity, they have distinct ligand-binding specificities.

In this study, we explore how human neutrophils recognize GBS and differentiate between live and dead bacteria. We show that neutrophils produce significantly higher levels of IL-8, a chemokine that attracts neutrophils, and ROS in response to live, but not killed, GBS. This enhanced response is mediated by the simultaneous activation of Toll-like receptor 8 (TLR8) and formyl peptide receptors (FPRs). Specifically, we show that FPR1 and FPR2 each recognize distinct formylated peptides released by live bacteria, working synergistically with TLR8-mediated detection of bacterial RNA. Our findings support the idea that formylated peptides represent an important class of vita-PAMPs that drive high-level IL-8 and ROS production, contributing to the neutrophil response to viable GBS.

## Materials and methods

### Bacterial strains

GBS WT strain H36B serotype Ib (21), *K. pneumoniae* AC133 (22) and *Staphylococcus aureus* USA300 (23) were grown in, respectively, Todd-Hewitt broth, Luria Bertani broth and Tryptic Soy broth (all from Oxoid) to the mid-late log phase. In selected experiments, GBS were grown in the presence of a sub-inhibitory concentration of the formyl peptidase inhibitor actinonin (32μg/ml; Sigma-Aldrich). After growth, bacteria were washed twice and resuspended to the desired concentration in non-pyrogenic PBS (0.01 M phosphate, 0.15 M NaCl [pH 7.4]; Euroclone) before addition to neutrophil suspensions. To obtain preparations of heat-killed bacteria (HK-GBS), GBS were grown in Carey’s chemically defined medium (24) to the late log phase, washed three times, and resuspended in non-pyrogenic PBS. Bacteria were killed by heating at 80 °C for 45 min, followed by extensive washing with distilled water and lyophilization. One μg of the heat-killed bacteria preparation corresponds to approximately 1 × 10^6^ CFUs. In some experiments, bacteria were killed by antibiotic treatment or by exposure to ultraviolet light. For antibiotic-induced killing, bacteria were treated with 1, 000 UI of penicillin and 1 mg/ml of streptomycin for 2h. For UV-induced killing, bacteria were exposed an UV lamp in quartz containers for 30 min. Bacterial killing was confirmed by colony counts. Total DNA and RNA were extracted from GBS exactly as described (25).

### GBS signal peptides

Fifteen GBS signal peptide sequences were identified in the Signal Peptide Database (http://www.signalpeptide.de). Custom-made peptides were synthesized by Genscript Corp. with a purity of ≥95%. The sequence of these peptides has been previously reported (26).

### Isolation and stimulation of human neutrophils

Peripheral blood samples were provided by the Transfusion Medicine Unit of the University Hospital Gaetano Martino, Messina, Italy. Samples were obtained from healthy volunteers providing their written informed consent according to the General Data Protection Regulation (EU) 2016/679 (GDPR). Human neutrophils were isolated from the whole blood of healthy donors using Ficoll-Paque Premium (Sigma-Aldrich) density gradient centrifugation as previously described (26). Staining with May/Grunwald/Giemsa showed that ~95% of isolated cells were morphologically mature neutrophils (bands and segmented). After isolation, neutrophils were suspended and seeded in 96 well plates (5 x 10^5^ cells per well) in 0.2 ml in RPMI 1060 supplemented with 10% FBS (both from Euroclone) and stimulated with heat killed or live bacteria at the indicated concentrations (μg/ml) or multiplicities of infection (MOI), respectively. After a 1h incubation at 37 °C in 5% CO_2_, penicillin (250 IU/ml) and streptomycin (250 µg/ml) were added to kill extracellular bacteria. Control wells were stimulated with *Escherichia coli* K12 ultrapure LPS (InvivoGen). The contribution of FPRs and TLRs to IL-8 and ROS production induced by live bacteria was evaluated by pretreating neutrophils for 1 hour at 37 °C in 5% CO_2_ with the selective FPR2 antagonist WRW4 (Abcam), the pan-FPR inhibitor Boc-2 (GenScript), the selective TLR8 inhibitor CU-CPT9a (InvivoGen) or the selective TLR2 inhibitor TL2-C29 (InvivoGen) at the indicated concentrations prior to stimulation with heat-killed (HK) or live bacteria. In another set of experiments, neutrophil stimulation with HK-GBS or LPS was preceded by a 1-h preincubation at 37 °C in 5% CO_2_ with the indicated concentrations of the prototypical FPR1 and FPR2 agonists f-MIFL (GenScript) or WKYMVM (Abcam), respectively, or with various concentrations of formylated peptides synthesized based on GBS signal peptide sequences (26), as indicated above. Cell culture supernatants were collected at 24 h and stored at -80 °C for cytokine measurements. Interleukin-8 (IL-8), tumor necrosis factor alpha (TNF-α), and interleukin-6 (IL-6) concentrations were determined in duplicate using the following murine enzyme-linked immunosorbent assay (ELISA) kits according to the manufacturer’s recommendations (R&D Systems): Human IL-8 DuoSet ELISA (DY208), Human TNF-alpha DuoSet ELISA (DY210) and Human IL-6 DuoSet ELISA (DY206). The lower detection limits of these assays were 31, 16 and 9 pg/ml, respectively.

### Gene expression

Neutrophils (3 x 10^6^ cells per well in 0.5 ml of RPMI supplemented with 10% FCS) were seeded in 24-well plates and stimulated with heat killed or live bacteria grown to the mid-log phase at the indicated concentrations or multiplicities of infection (MOI), respectively. After incubation for 2 h at 37 °C with 5% CO_2_, RNA extraction was conducted by using RNeasy Mini Kit (QIAGEN), according to the manufacturer’s protocols. RNA was then reverse transcribed to cDNA, using the iScript cDNA Synthesis kit (Bio-Rad, Hercules, CA, USA). Human gene expression for the IL-8 and beta-actin genes was measured by quantitative real-time PCR (qPCR) using a CFX96 Touch Real-Time PCR Detection System (Bio-Rad) and pre-validated TaqMan probes (IDs Hs00174103_m1 and hs01060665_g1, respectively, all from Applied Biosystems). Relative gene expression was carried out with the comparative CT method (2^−ΔΔCT^).

### Neutrophil migration assay

Neutrophil chemotaxis was determined using ChemoTX multiwell chambers (NeuroProbe) with 3 μm pore size filters according to the manufacturer’s protocol. Briefly, peptides or vehicle were diluted to the desired concentration in RPMI medium supplemented with 0.1% BSA (w/v) and 315 μl were added to the lower chamber of each well. Neutrophil suspensions (1 × 10^5^ per well in 57 μl of RPMI plus 0.1% BSA) were placed on top of the polycarbonate filter above each well and allowed to migrate for 90 min at 37 °C with 5% CO_2_. The assay was terminated by detaching and wiping the filter to remove non-migrated cells from the filter top. The number of transmigrated cells was determined using BD TruCount tubes (BD Biosciences).

### Reactive oxygen species measurement

Bacteria-induced reactive oxygen species (ROS) production by neutrophils was measured using the CellROX Deep Red Flow Cytometry Assay Kit (Thermo Fisher Scientific), exactly as previously described (26). Briefly, neutrophils (5 × 10^5^ per well in 0.2 ml of RPMI supplemented with 10% FCS) were seeded in microtiter plates and stimulated for 30 min with bacteria and/or with the indicated formylated peptides. After stimulation, samples were stained with the CellROX fluorescent reagent (5 μM) for 30 min at 37 °C in 5% CO_2_. Cells were washed, fixed with 3.7% formaldehyde for 15 min, and analyzed with a BD FACS Canto II instrument.

### Neutrophil killing assay

For killing assays, neutrophils (5 x 10^5^ cells/well) were infected with live GBS (4x10^5^ CFU/well) and incubated at 37 °C with 5% CO_2_ for different times. After three washes with PBS and killing of extracellular bacteria through the addition of medium containing gentamicin (100 mg/ml), cells were lysed with 0.025% Triton X-100 to release intracellular bacteria. Recovered bacteria were plated on agar plates for CFU counts.

### Statistical analyses

Differences in cytokine and gene expression levels and ROS production were assessed by the Mann-Whitney test. Differences were considered statistically significant when P values were less than 0.05 (P < 0.05). Statistical analyses were performed with GraphPad Prism 5.0 (GraphPad Software, Inc., San Diego, CA).

## Results

### Live GBS induces high levels of IL-8 and ROS in an FPR-dependent manner

**Figure 1:**
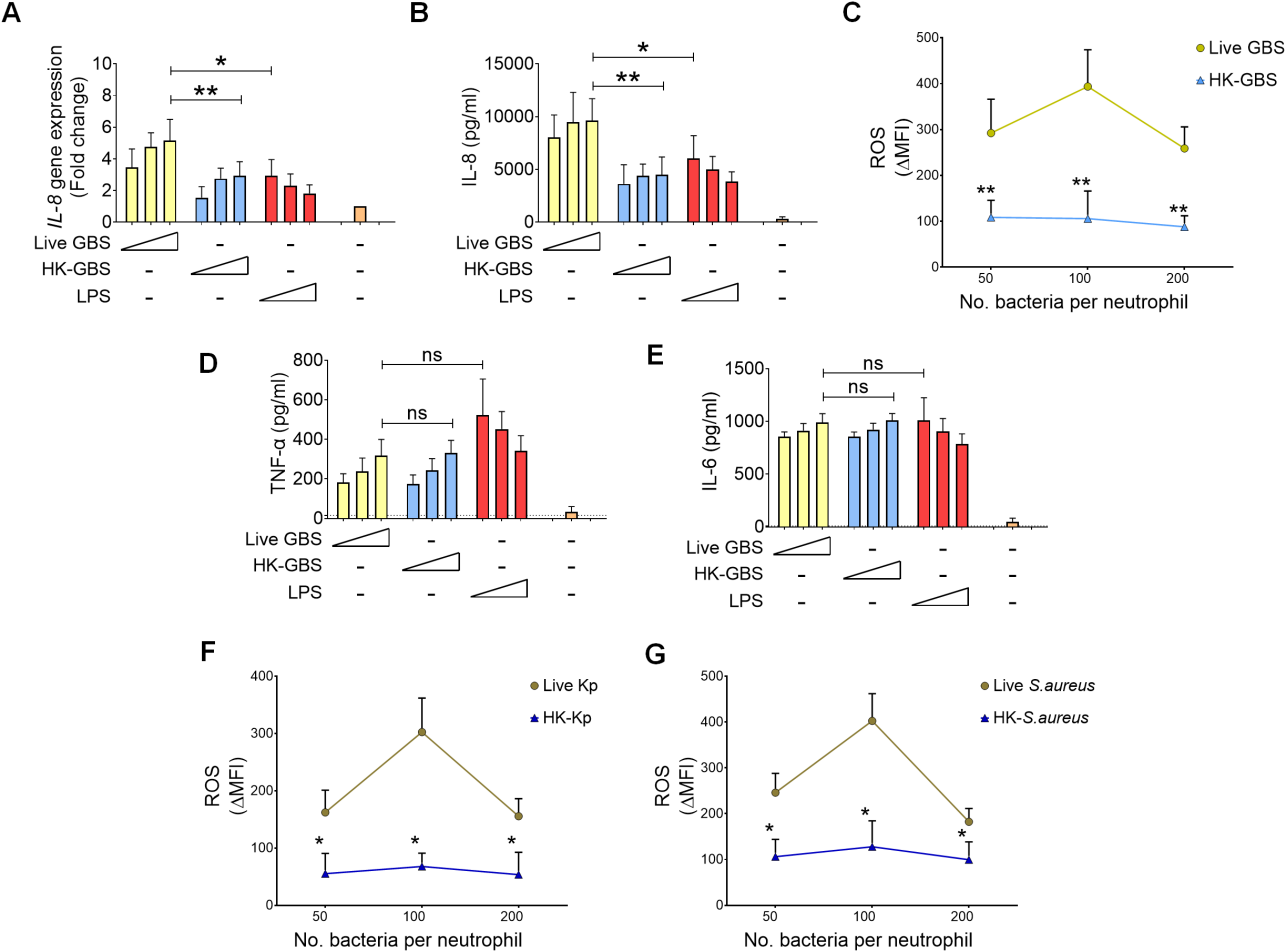
Live, but not killed, GBS induce high-level IL-8 and ROS release. IL-8 gene expression **(A)** and IL-8 protein concentrations **(B)** in human neutrophils stimulated live or heat killed GBS (HK-GBS). Live GBS was used at MOIs of 2, 5, and 10. HK-GBS was used at concentrations of 10, 25 and 50 µg/mL corresponding approximately to 4, 10 and 20 bacterial cells per neutrophil. *Escherichia coli* lipopolysaccharide (LPS; 10, 100, and 1000ng/mL) was included as a positive control, while unstimulated neutrophils served as a negative control. Panel **(C)** shows Δ median fluorescence intensities (ΔMFI) values obtained using the CellROX assay, a measure of reactive oxygen species concentrations, in neutrophils stimulated with the indicated numbers of live GBS or HK-GBS per neutrophil. TNF-α **(D)** and IL-6 **(E)** concentrations in neutrophils stimulated with live GBS, HK-GBS or LPS at the doses indicated in the legend to panels **(A)** and **(B)**. Panels **(F)** and **(G)** show ROS release, expressed as ΔMFI values, in neutrophils stimulated with the indicated numbers of live or HK-*Klebsiella pneumoniae* (Kp) and *Staphylococcus aureus* (*S. aureus*). Data are expressed as means ± standard deviations from five independent experiments, each performed in duplicate. *p < 0.05, **p < 0.01, as determined by the Mann-Whitney test; ns, not significant.

In previous studies using mouse models, we demonstrated that live, but not killed, bacteria stimulate neutrophils to produce high levels of Cxcl2, a chemokine that recruits additional neutrophils through a positive feedback loop (27). This mechanism is crucial for signaling the presence of live bacteria and mounting an effective immune response (26, 27). Here, we investigated whether human neutrophils exhibit a similar response, focusing on IL-8, which is functionally homologous to murine Cxcl1 and Cxcl2 (28). In human neutrophils, both live and killed GBS induced IL-8 release in a dose-dependent fashion. However, live GBS induced significantly higher maximal levels of *IL-8* gene expression (**Figure 1A**) and protein release (**Figure 1B**) than those induced by heat-killed GBS or *Escherichia coli* lipopolysaccharide (LPS), the latter serving as a positive control. Similarly, live GBS was substantially more effective than heat-killed GBS in inducing production of reactive oxygen species (ROS), a key antimicrobial response (**Figure 1C**). Weak induction of ROS was also observed with bacteria killed by antibiotics or ultraviolet light, ruling out the possibility that reduced ROS production from heat-killed bacteria is due to inactivation of a heat-labile PAMP of GBS (**Supplementary Figure S1**). In contrast, the maximal levels of other cytokines, such as TNF-α (**Figure 1D**) and IL-6 (**Figure 1E**), did not differ significantly between live and killed bacteria, although approximately twice as many killed bacteria were needed to reach maximal levels. To investigate whether the enhanced ability of live bacteria to induce IL-8 and ROS is specific to GBS, we examined the responses to *Klebsiella pneumoniae* and *Staphylococcus aureus*, two other common bacterial pathogens. Similar to GBS, live *K. pneumoniae* and *S. aureus* induced significantly higher levels of ROS and IL-8, compared to their killed counterparts (**Figures 1F, G**; **Supplementary Figure S2**). Collectively, these findings suggest that live, but not killed, bacteria are potent inducers of IL-8 and ROS in human neutrophils.

**Figure 2:**
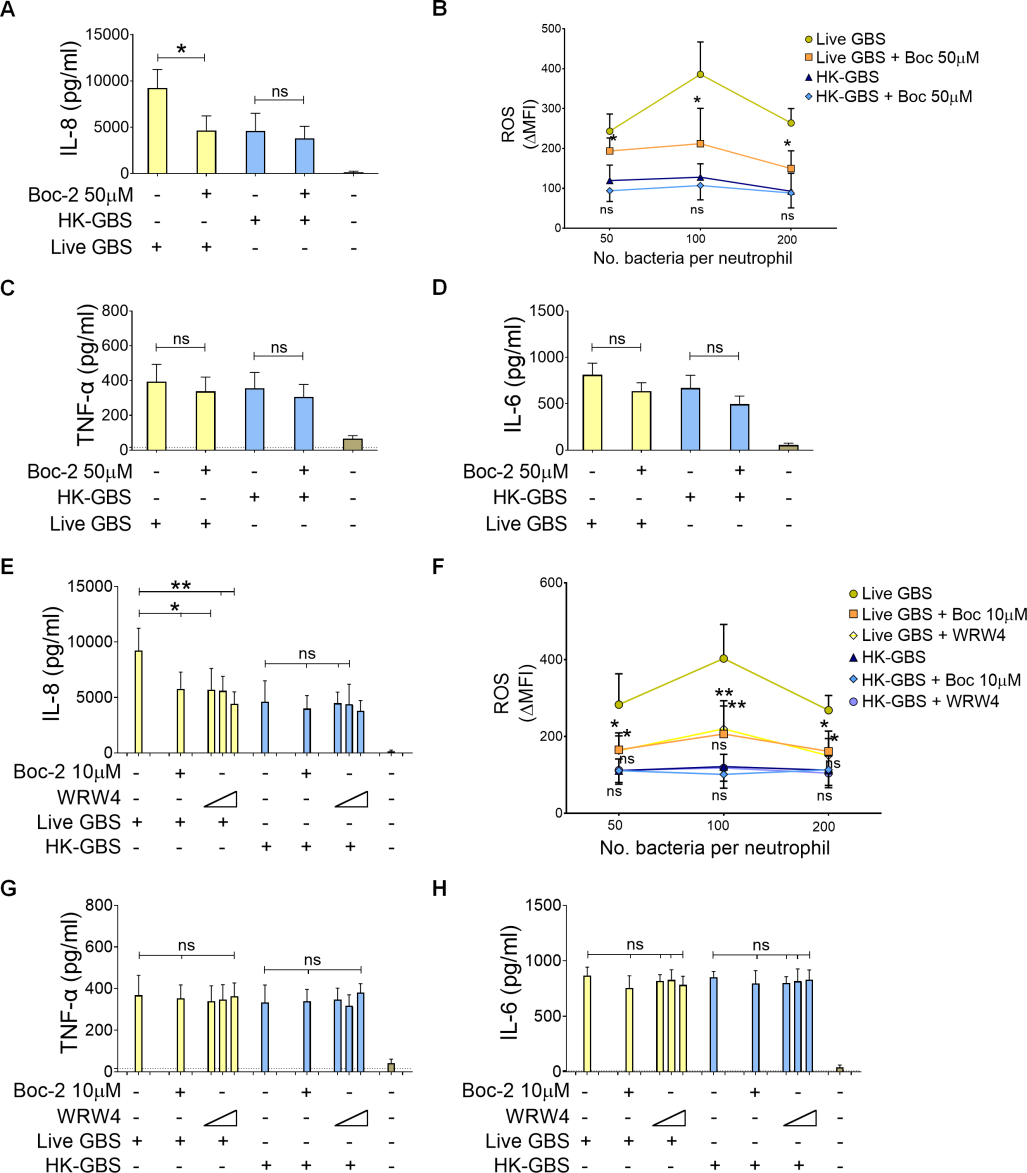
Role of formyl peptide receptors in GBS-induced cytokine release**. (A, C, D)** Effect of Boc-2, a formyl peptide receptor antagonist, on GBS-induced IL-8, TNF-α and IL-6 production. Neutrophils were exposed to Boc-2 (50µM) and then stimulated with live GBS (MOI of 5) or heat-killed GBS (HK-GBS, 25μg/ml) as indicated in the legend to **Figure 1A**. **(B)** Effect of Boc-2 (50µM) on reactive oxygen species, as determined with the CellROX assay, after neutrophil stimulation with live or HK-GBS as indicated in the legend to **Figure 1C**. Release of IL-8 **(E)**, reactive oxygen species **(F)**, TNF-α **(G)** and IL-6 **(H)** in neutrophils stimulated with live (MOI of 5) or killed (25μg/ml) GBS. Cells were pre-treated with Boc-2 (10μM) or WRW4 (1, 5, and 10μM) to selectively inhibit FPR1 and FPR2, respectively. Data are expressed as means ± standard deviations from five independent experiments, each performed in duplicate. *p < 0.05 and **p < 0.01, as determined by the Mann-Whitney test; ns, not significant.

Next, we hypothesized that the high-level induction of IL-8 and ROS by live bacteria might be linked to the release of formylated peptides produced during bacterial protein synthesis, previously implicated in Cxcl2 induction in murine neutrophils (26). To test this hypothesis, we examined the effects of blocking receptors that sense formylated peptides, specifically formyl peptide receptor 1 and 2 (FPR1 and FPR2). Pretreatment of neutrophils with 50 μM Boc-2, which blocks both FPR1 and FPR2 (29), resulted in significant reductions in IL-8 (**Figure 2A**) and ROS (**Figure 2B**) responses to live, but not to killed, GBS. In contrast, TNF-α and IL-6 production induced by either live or killed bacteria was unaffected (**Figures 2C, D**). When neutrophils were pretreated with 10 μM Boc-2, which specifically inhibits FPR1 at this concentration, we also observed a significant reduction in IL-8 (**Figure 2E**) or ROS (**Figure 2F**) in response to live GBS, again with no impact on responses to killed GBS (**Figures 2E, F**). Similarly, pretreatment with WRW4, a selective antagonist of FPR2 (30), caused partial but significant inhibition of IL-8 (**Figure 2E**) and ROS (**Figure 2F**) release in response to live, but not killed, bacteria, with no effect on TNF-α or IL-6 production (**Figures 2G, H**). These findings suggest that both FPR1 and FPR2 contribute non-redundantly to the robust induction of IL-8 and ROS by live bacteria, whereas other cytokine responses (e.g., TNF-α, IL-6) are independent of this pathway.

**Figure 3:**
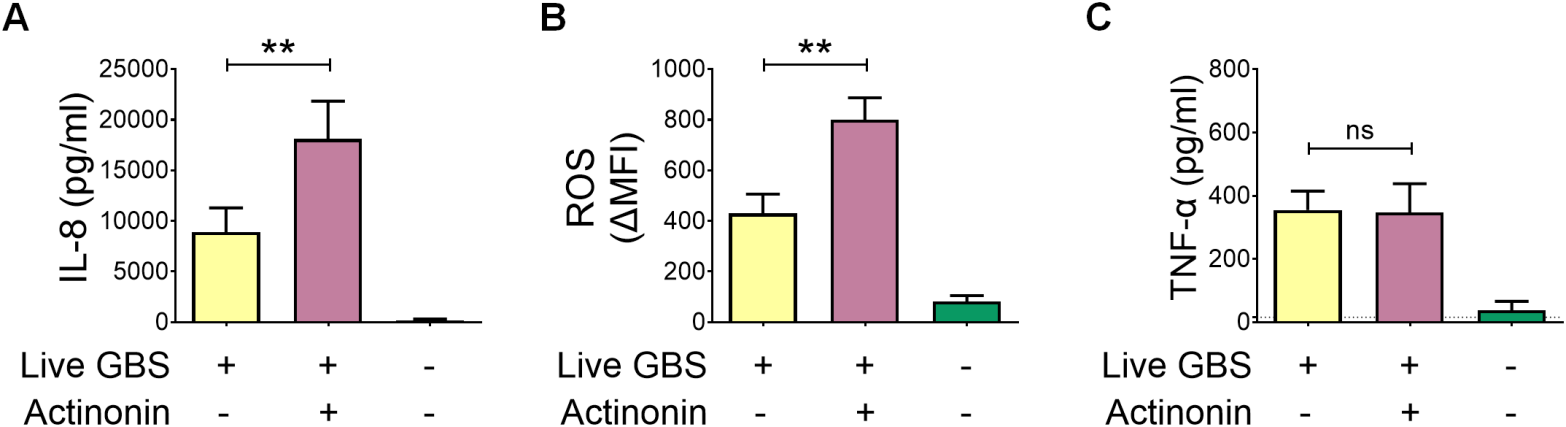
Peptide deformylase inhibition enhances IL-8 and ROS release in response to GBS. Effect of actinonin, a peptide deformylase inhibitor, on IL-8 **(A)**, reactive oxygen species **(B)** and TNF-α **(C)** release by neutrophils stimulated with live GBS at MOIs of 5 **(A, C)** or 100 **(B)**. Neutrophils were stimulated with bacteria grown in the presence or in the absence of a sub-inhibitory concentration of actinonin (32μg/ml). Data are expressed as means ± standard deviations from five independent experiments, each performed in duplicate. **p < 0.01, as determined by the Mann-Whitney test; ns, not significant.

FPR1 and FPR2 are promiscuous receptors that are capable of recognizing not only formylated peptides but also compounds with other chemical structures. Therefore, we specifically investigated the involvement of formylated peptides in promoting FPR-dependent release of IL-8 and ROS in the context of stimulation with live GBS. To this end, we used actinonin, an antibiotic that promotes the accumulation of formylated peptides through its ability to inhibit peptide deformylase, the enzyme that removes terminal N-formyl groups from these peptides (31). GBS was grown in the presence of a sub-inhibitory concentration of actinonin and used to stimulate peripheral blood neutrophils. Remarkably, actinonin-grown GBS induced significantly higher IL-8 and ROS responses in neutrophils compared to bacteria grown in the absence actinonin (**Figures 3A, B**). In contrast, no differences were observed in the ability of bacteria to induce TNF-α, regardless of whether actinonin was present (**Figure 3C**). Collectively, these findings suggest that the interaction between bacterial formylated peptides and FPR1/2 is crucial for the ability of live bacteria to stimulate high levels of IL-8 and ROS production in human neutrophils.

### Identification of immunostimulatory formylated peptides in GBS

**Figure 4:**
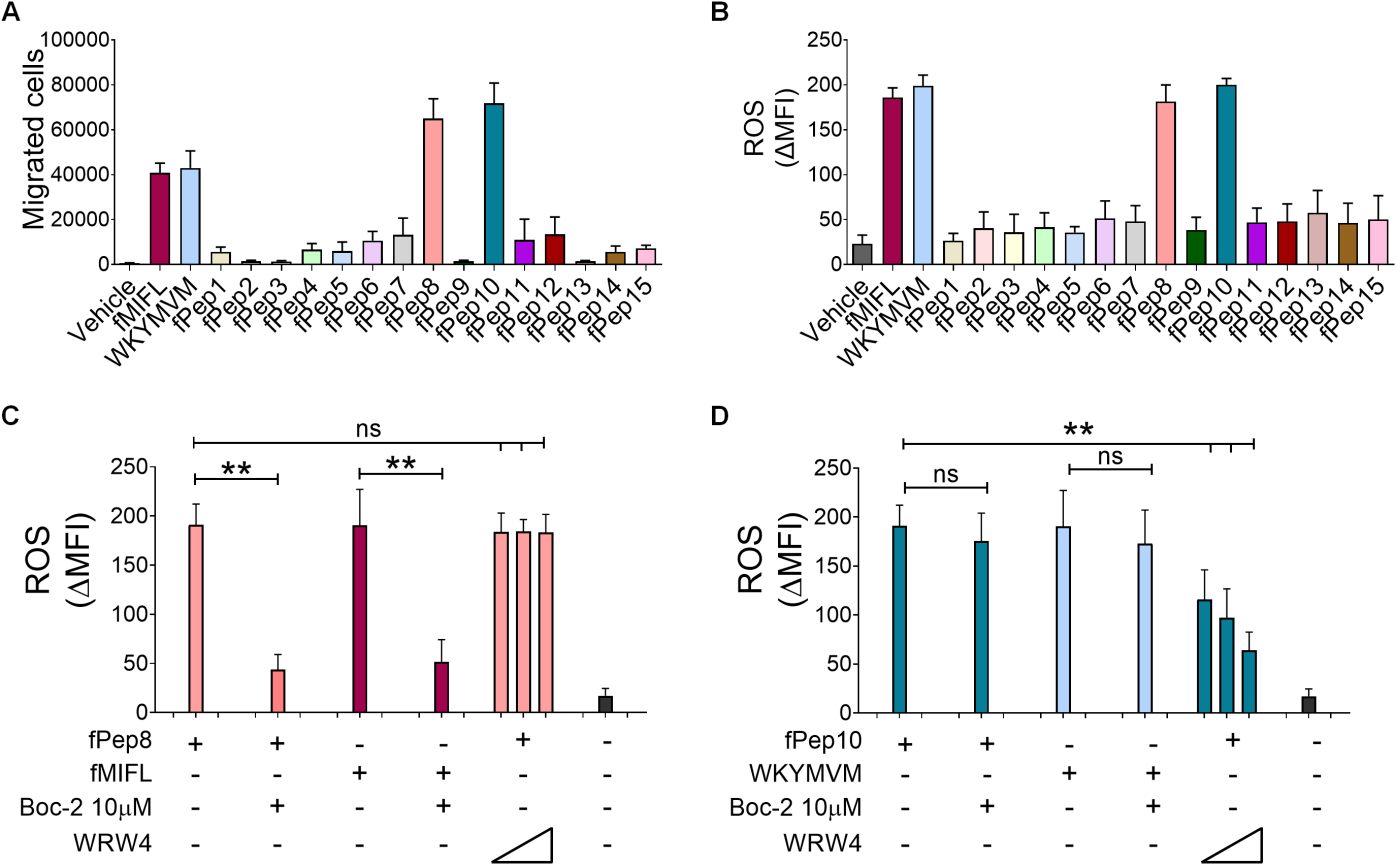
Induction of reactive oxygen species by formylated peptides from GBS. **(A)** Neutrophils were allowed to migrate towards each of 15 formylated peptides synthesized after signal peptide sequences from GBS (fPep1-15, 1µM). The formylated peptides fMIFL and WKYMVM (both at 1µM) were used as positive controls. Vehicle, 0.2% DMSO. **(B–D)** Release of reactive oxygen species in neutrophils stimulated with GBS peptides. Neutrophils were pretreated with the FPR1 inhibitor Boc (10µΜ) or the FPR2 inhibitor WRW4 (1, 5, and 10μM) before stimulation with the GBS peptides fPep8 **(C)** and fPep10 **(D)**. Data are expressed as means ± standard deviations from five independent experiments, each performed in duplicate. **p < 0.01, as determined by the Mann-Whitney test; ns, not significant.

We next sought to identify the formylated peptides responsible for augmenting IL-8 and ROS responses to GBS. Signal peptides are N-terminal sequences that direct protein transport across membranes. In previous work, we synthesized hexapeptides based on known secretory signal sequences and demonstrated that selected formylated signal peptides from GBS can activate FPRs in murine neutrophils (26). Here, we assessed the ability of these peptides to activate human neutrophils, using as positive controls fMILF and WKYMVM, which are prototypical FPR1 and FPR2 agonists, respectively. Among 15 peptides tested, two—fPep8 and fPep10—promoted strong *in vitro* chemotactic responses (**Figure 4A**) and stimulated reactive oxygen species (ROS) production in human neutrophils (**Figure 4B**). Notably, ROS production and chemotaxis triggered by fPep8 were inhibited by 10 μM Boc-2, a concentration that selectively blocks FPR1, whereas the FPR2-specific antagonist WRW4 had no effect (**Figure 4C**). In contrast, the activity of fPep10 was abrogated by WRW4 but not affected by Boc-2 (**Figure 4D**). Moreover, co-treatment with fPep8 and fPep10 did not significantly enhance chemotaxis compared to each peptide administered individually (**Supplementary Figure S3**), indicating the absence of additive or synergistic effects. These findings suggest that fPep8 and fPep10 activate neutrophils through distinct receptors, with fPep8 engaging FPR1 and fPep10 engaging FPR2.

### TLR8 is involved in cytokine induction by both live and dead bacteria

Toll-like receptor 8 (TLR8) is highly expressed in the endosomes of myeloid cells, including neutrophils, where it can sense the presence of RNA derived from a wide range of pathogens, including viruses, bacteria, and protozoa (18, 19). TLR8 has recently emerged as a dominant sensor by which human macrophages sense the presence of bacteria, including GBS (32). It has been proposed, in addition, that TLR-8 mediates the ability of antigen-presenting cells to distinguish viable from dead bacteria (17). Since little is known of the functions of TLR8 in neutrophils, we assessed its role in these cells using CU-CPT9a, a recently developed, highly specific inhibitor that stabilizes the TLR8 dimer in its resting state, thereby preventing its activation (33). For comparative purposes, we also employed TL2-C29 (34), a specific inhibitor of TLR2, a Toll-like receptor thought to be involved in cytokine induction by Gram-positive bacteria in macrophages (35).

**Figure 5:**
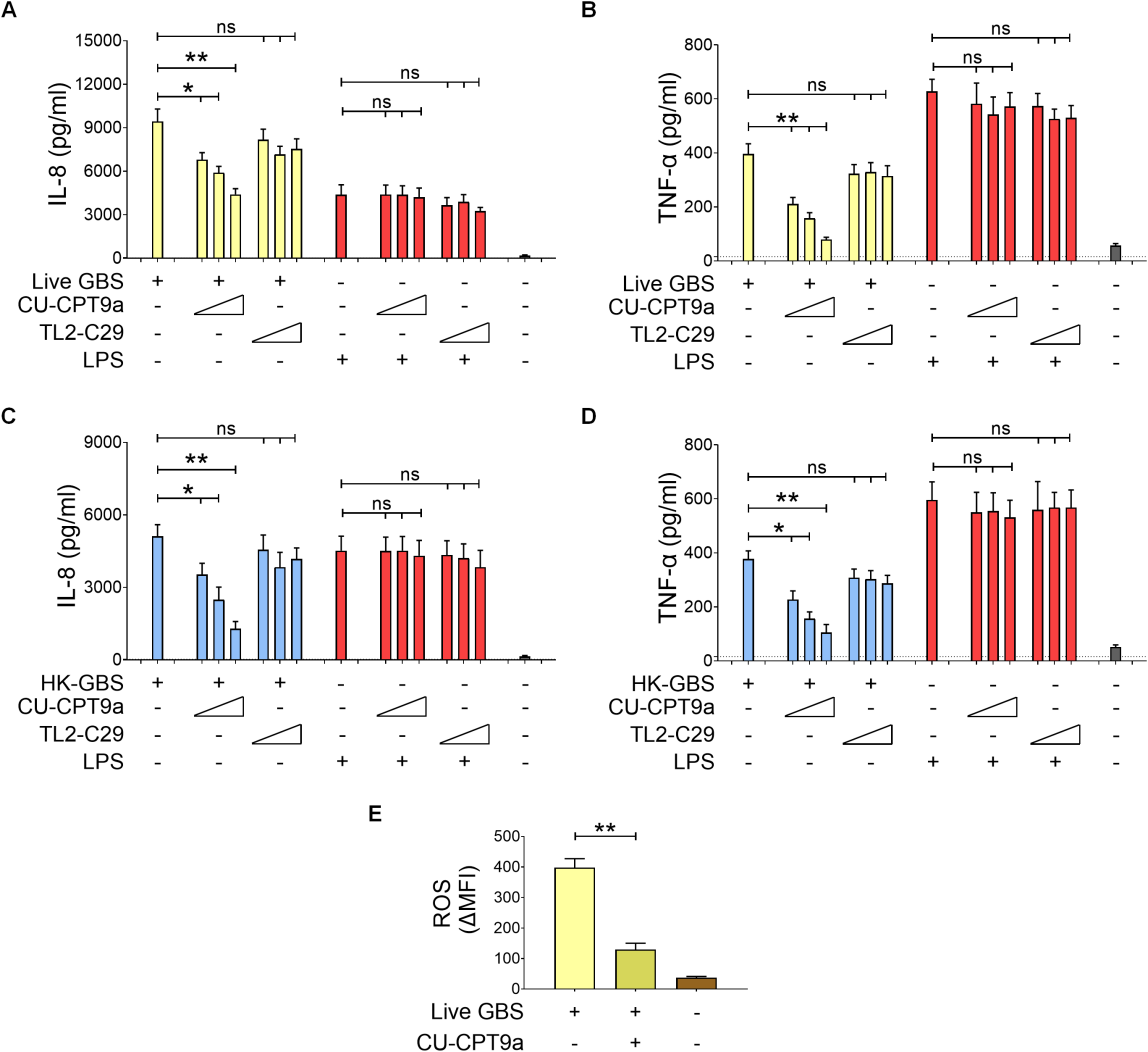
TLR8 is required for GBS-induced cytokine production. Neutrophils were treated with the TLR8 inhibitor CU-CPT9a (0.75, 1.50 and 3.00μM) or the TLR2 inhibitor TL2-C29 (5, 10 and 25μM) before stimulation with live [MOI of 5, **(A, B)**] or heat-killed GBS [25 μg/ml, **(C, D)**]. IL-8 **(A, C)** and TNF-α **(B, D)** were measured in 24h culture supernatants. *Escherichia coli* lipopolysaccharide (LPS; 10 ng/mL) was included as a positive control. **(E)** Effect of pretreatment with the TLR8 CU-CPT9a inhibitor (3μM) on the release of reactive oxygen species after stimulation with live GBS (MOI 100). Data are expressed as means ± standard deviations from five independent experiments, each performed in duplicate. *p < 0.05 and **p < 0.01, as determined by the Mann-Whitney test; ns, not significant.

**Figure 6:**
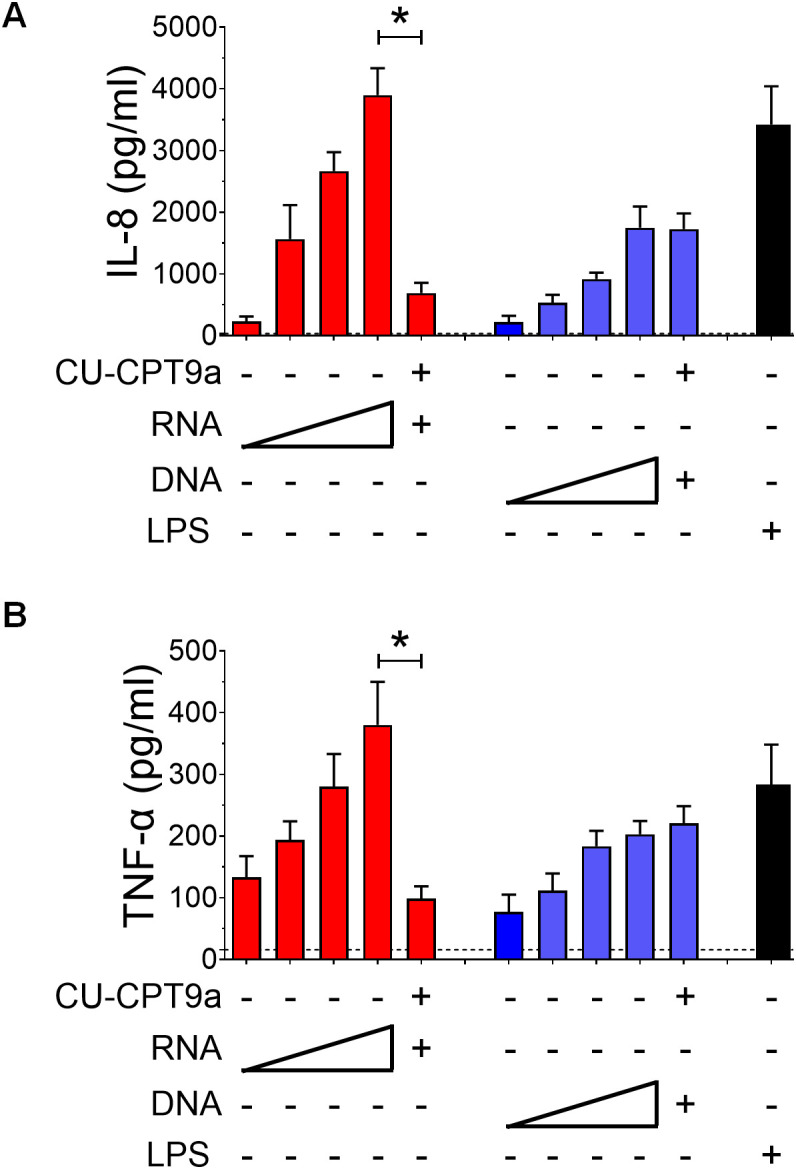
Cytokine responses to GBS by nucleic acids. IL-8 **(A)** and TNF-α **(B)** responses to stimulation with graded doses of GBS nucleic acids (0.01, 0.1, 1 and 10 µg/ml). In experiments involving pretreatment with the TLR8 inhibitor CU-CPT9a (3 μΜ), neutrophils were then stimulated with 10 µg/ml of nucleic acids (RNA or DNA). Data are expressed as means ± standard deviations from three independent experiments, each performed in duplicate. *p < 0.05, as determined by the Mann-Whitney test.

Pretreatment of neutrophils with CU-CPT9a, but not TL2-C29, resulted in marked, dose-dependent reduction in IL-8 and TNF-α production in response in both live (**Figures 5A, B**) and heat-killed bacteria (**Figures 5C, D**) without affecting responses to LPS, used as a control. In addition, CU-CPT9a also markedly reduced ROS induction by live GBS (**Figure 5E**). Similar results were observed when using *K. pneumoniae* and *S. aureus* as stimuli (**Supplementary Figure S4**). Furthermore, pharmacological inhibition of TLR8, FPR1 or FPR2 significantly decreased live GBS killing at early time points, demonstrating that activation of these receptors enhances bacterial killing (**Supplementary Figure S5**). Since TLR8 is known to recognize RNA, we compared the ability of GBS RNA to stimulate human neutrophils with that of DNA, which activates TLR9, another endosomal receptor (35). We found that RNA was significantly more potent than DNA in inducing cytokine production, and that RNA-induced cytokine release was strongly inhibited by the TLR8-specific inhibitor CU-CPT9a (**Figure 6**). Taken together, these results indicate that TLR8 plays a critical role in bacterial recognition and the promotion of antimicrobial responses in human neutrophils through its ability to recognize bacterial RNA. Notably, TLR8 is involved in responses to both live and heat-killed GBS, whereas FPR receptors are specifically required for the high-level production of IL-8 and ROS induced by live, but not killed, bacteria.

### fPep8 and fPep10 synergize with TLR agonists in the induction of IL-8

**Figure 7:**
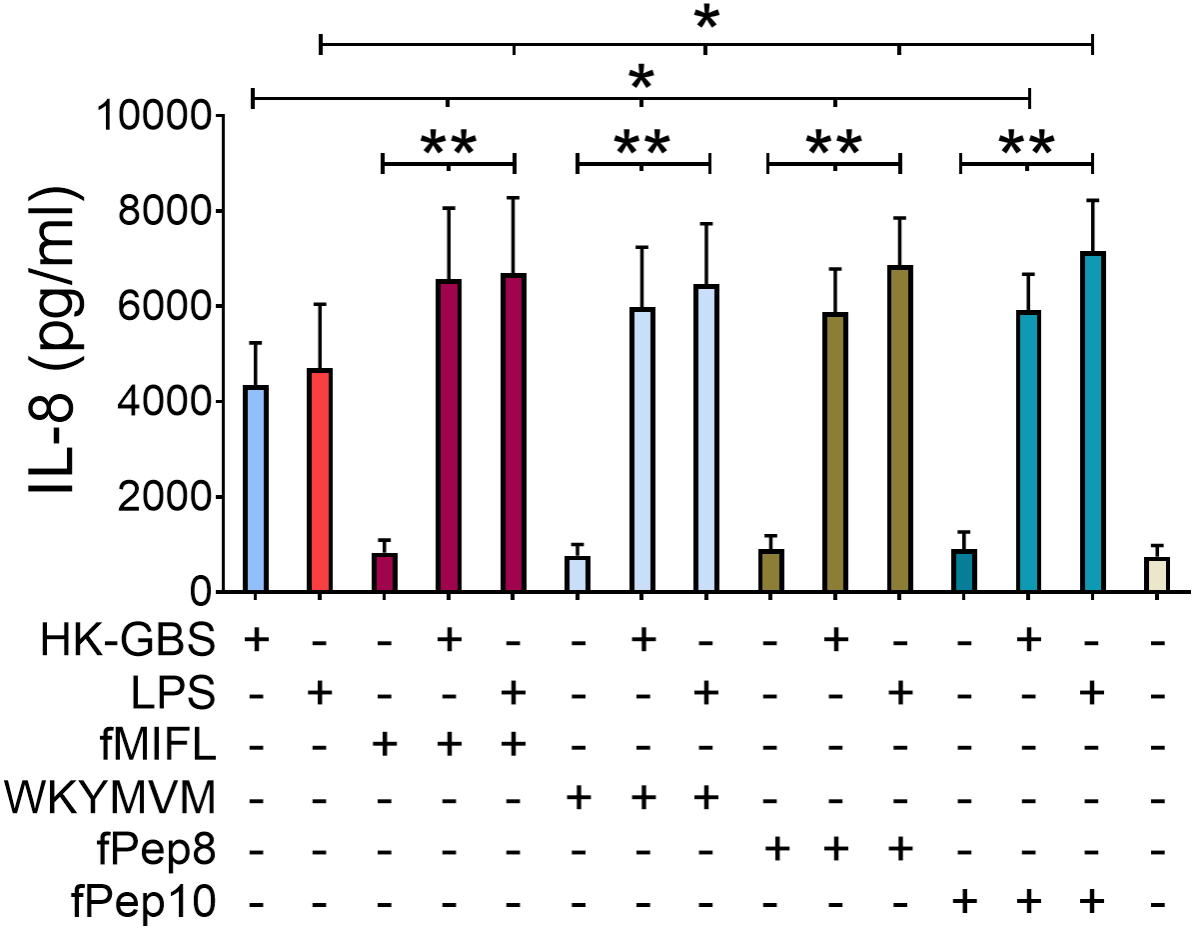
GBS formylated peptides cooperate with TLR agonists in IL-8 production. Release of IL-8 in neutrophils stimulated with combinations of TLR and FPR agonists. Neutrophils were pre-treated with formylated peptides (fMIFL, WKYMVM, fPep8 or fPep10, all at a 1 μΜ concentration) for 15 min before the addition of heat-killed GBS (HK-GBS, 25μg/ml) or LPS (10) ng/ml. Data are expressed as means ± standard deviations from five independent experiments, each performed in duplicate. *p < 0.05 and **p < 0.01, as determined by the Mann-Whitney test.

Next, we hypothesized that GBS formylated peptides might synergize with TLR8 and other TLR agonists to enhance IL-8 production in neutrophils. None of the formylated peptides tested, including GBS formylated peptides and the well-characterized FPR agonists f-MIFL and WKYMVM, were able to independently induce IL-8 production (**Figure 7**). However, both f-MIFL and WKYMVM significantly enhanced IL-8 production in response to TLR agonists such as LPS or heat-killed bacteria (**Figure 7**). Similarly, the GBS-derived peptides fPep8 and fPep10 also potentiated IL-8 induction in the presence of TLR agonists, indicating a synergistic interaction occurring through the engagement of either FPR1 or FPR2 in conjunction with TLR signaling. Collectively, these findings show that activation of FPR1 or FPR2 alone is insufficient to drive IL-8 production. However, concurrent activation of TLRs and either FPR1 or FPR2 synergistically promotes robust IL-8 release in human neutrophils.

## Discussion

Despite their key role in host defense against GBS, the mechanisms by which neutrophils respond to the presence of these bacteria are unclear. Upon encountering GBS, neutrophils produce a range of cytokines and chemokines. While individual neutrophils produce lower levels of cytokines compared to mononuclear phagocytes, their sheer numbers make them major contributors to overall cytokine production (21). Because of this, neutrophils play a major role in promoting the recruitment of additional inflammatory cells in the initial phases of infection (7), also amplifying their own recruitment through the secretion of neutrophil-attracting chemokines, particularly Cxcl2 (23, 27). In this study, we show that human neutrophils predominantly sense GBS via Toll-like receptor 8 (TLR8), which is highly expressed in the endosomes of human myeloid cells, including neutrophils, as a functional cleavage product (32). To investigate the role of TLR8, we used CU-CPT9a, a selective chemical antagonist previously shown to fully block cytokine production in monocytes stimulated with TLR8, but not TLR2 or TLR4, agonists (33). Upon challenge with different bacterial pathogens, the TLR8 inhibitor significantly reduced the production of all tested pro-inflammatory and chemotactic cytokines, as well as reactive oxygen species (ROS). In contrast, the TLR2 inhibitor TL2-C29 had no measurable effect on cytokine production induced by live bacteria. Our results align with our earlier work demonstrating that TLR2 is dispensable for mouse macrophage responses to whole GBS, although concentrated lipoprotein-containing bacterial supernatants can induce TLR2-dependent stimulation (36). On the other hand, in human monocytes and macrophages TLR2 ligands can suppress, rather than enhance, TLR8-driven cytokine production (32). Given that TLR8 recognizes bacterial RNA, we examined the cytokine-inducing capacity of purified GBS RNA and found that it potently activates human neutrophils in a TLR8-dependent fashion. Overall, our data strongly suggest that TLR8-mediated recognition of RNA is a dominant mechanism of neutrophil activation by GBS and are consistent with prior studies using human monocytes and monocyte-derived macrophages (15) or whole blood (37). However, due to the known limitations of pharmacological inhibitors, such as potential off-target effects and suboptimal target engagement, future studies will be required to further validate the role of TLR8 in immune recognition of GBS in human neutrophils. Indeed, the overall contribution of TLR8 to host defense against GBS and other encapsulated bacteria remains unclear and warrants further investigation. Unfortunately, suitable small animal models are lacking, as murine TLR8 differs significantly from its human counterpart and is not responsive to the same agonists (38). Nonetheless, murine TLR13, a bacterial RNA sensor absent in humans but functionally analogous to TLR8, plays an important role in host defense against GBS (39), *S. pneumoniae* (40), and *K. pneumoniae* (22). Together, findings from human cells and mouse models support that bacterial RNA is a critical target for innate immune recognition by mononuclear and polymorphonuclear phagocytes. Our findings extend previous observations that neutrophils respond more robustly to live bacteria than to killed ones, particularly with respect to ROS production and chemokine release (26, 41). Although live bacteria induced cytokines approximately twice as efficiently per cell, as compared to killed bacteria, maximal responses were similar for most cytokines except IL-8, and heat-killed bacteria remained weak inducers of ROS even at high doses. We show here that full IL-8 release and ROS production in response to live bacteria require the combined engagement of FPRs and TLR8. Of note, FPR1 and FPR2 appear to selectively recognize distinct GBS-derived formylated peptides, as shown in murine models genetically lacking these receptors (26) and confirmed here with specific pharmacological inhibitors in human neutrophils. Specifically, we identified fpep8, the N-terminal hexapeptide signal sequence of the CAMP factor (42), as selective FPR1 agonist, and fPep10, from the FbsA virulence factor signal sequence (43), as a selective FPR2 agonist in both mouse and human neutrophils. However, it is likely that, beyond the two formylated peptides identified here, many others also contribute to neutrophil activation via FPRs and further research is needed to characterize the full spectrum of FPR agonists produced by GBS.

In conclusion, our data show that human neutrophils produce significantly more IL-8 and ROS in response to live, but not heat-killed, bacteria by integrating signals originating from TLR8 and FPRs and that this multi-receptor system enables neutrophils to discriminate between inert microbial debris and viable bacterial threats. We propose that the requirement of multiple receptors (involving FPR1, FPR2 and TLR8) for high-level ROS and IL-8 production serves as a safeguard, ensuring that neutrophils are fully activated only when live bacteria pose an immediate threat.

## Data Availability

The datasets generated for this study areavailable on request to the corresponding author.

## References

[1] Farley MM . Group B streptococcal disease in nonpregnant adults. Clin Infect Dis. (2001) 33:556–61. doi: 10.1086/322696. PMID: 11462195

[2] Sambola A Miro JM Tornos MP Almirante B Moreno-Torrico A Gurgui M . Streptococcus agalactiae infective endocarditis: analysis of 30 cases and review of the literature, 1962-. Clin Infect Dis. (1998) 34:1576–84. doi: 10.1086/340538. PMID: 12032892

[3] Zi M Zhang Y Hu C Zhang S Chen J Yuan L . A literature review on the potential clinical implications of streptococci in gastric cancer. Front Microbiol. (2022) 13:1010465. doi: 10.3389/fmicb.2022.1010465. PMID: 36386672 PMC9643750

[4] Keogh RA Doran KS . Group B Streptococcus and diabetes: Finding the sweet spot. PloS Pathog. (2023) 19:e1011133. doi: 10.1371/journal.ppat.1011133. PMID: 36795681 PMC9934380

[5] Edwards MS Baker CJ . Group B streptococcal infections in elderly adults. Clin Infect Dis. (2005) 41:839–47. doi: 10.1086/432804. PMID: 16107984

[6] Verani JR McGee L Schrag SJ Division of Bacterial Diseases NCfIaRD Centers for Disease Control and Prevention (CDC) . Prevention of perinatal group B streptococcal disease--revised guidelines from CDC, 2010. MMWR Recomm Rep. (2010) 59:1–36 21088663

[7] Biondo C Mancuso G Midiri A Signorino G Domina M Lanza Cariccio V . The interleukin-1β/CXCL1/2/neutrophil axis mediates host protection against group B streptococcal infection. Infect Immun. (2014) 82:4508–17. doi: 10.1128/IAI.02104-14. PMID: 25114117 PMC4249330

[8] Urlichs F CPS . Neutrophil function in preterm and term infants. Neoreviews. (2004) 5:e417–29. doi: 10.1542/neo.5-10-e417

[9] Engle WA McGuire WA Schreiner RL Yu PL . Neutrophil storage pool depletion in neonates with sepsis and neutropenia. J Pediatr. (1988) 113:747–9. doi: 10.1016/s0022-3476(88)80394-9. PMID: 3270337

[10] Ugolini M Sander LE . Dead or alive: how the immune system detects microbial viability. Curr Opin Immunol. (2019) 56:60–6. doi: 10.1016/j.coi.2018.09.018. PMID: 30366275

[11] Rauh LW Schmidt R . Measles immunization with killed virus vaccine. Serum antibody titers and experience with exposure to measles epidemic. 1965. Bull World Health Organ. (2000) 78:226–31. doi: 10.1001/archpedi.1965.02090020234007. PMID: 10743294 PMC2560693

[12] Lauvau G Vijh S Kong P Horng T Kerksiek K Serbina N . Priming of memory but not effector CD8 T cells by a killed bacterial vaccine. Science. (2001) 294:1735–9. doi: 10.1126/science.1064571. PMID: 11721060

[13] Sander LE Davis MJ Boekschoten MV Amsen D Dascher CC Ryffel B . Detection of prokaryotic mRNA signifies microbial viability and promotes immunity. Nature. (2011) 474:385–9. doi: 10.1038/nature10072. PMID: 21602824 PMC3289942

[14] Blander JM Sander LE . Beyond pattern recognition: five immune checkpoints for scaling the microbial threat. Nat Rev Immunol. (2012) 12:215–25. doi: 10.1038/nri3167. PMID: 22362354

[15] Ehrnström B Beckwith KS Yurchenko M Moen SH Kojen JF Lentini G . Toll-like receptor 8 is a major sensor of group B. Front Immunol. (2017) 8:1243. doi: 10.3389/fimmu.2017.01243. PMID: 29042860 PMC5632357

[16] Maserumule C Passemar C Oh OSH Hegyi K Brown K Weimann A . Phagosomal RNA sensing through TLR8 controls susceptibility to tuberculosis. Cell Rep. (2025) 44:115657. doi: 10.1016/j.celrep.2025.115657. PMID: 40338743 PMC7618372

[17] Ugolini M Gerhard J Burkert S Jensen KJ Georg P Ebner F . Recognition of microbial viability via TLR8 drives T._FH_ cell differentiation and vaccine responses. Nat Immunol. (2018) 19:386–96. doi: 10.1038/s41590-018-0068-4. PMID: 29556002

[18] Nunes IV Breitenbach L Pawusch S Eigenbrod T Ananth S SChad P . Bacterial RNA sensing by TLR8 requires RNase 6 processing and is inhibited by RNA 2'O-methylation. EMBO Rep. (2024) 25:4674–92. doi: 10.1038/s44319-024-00281-9. PMID: 39363059 PMC11549399

[19] Ostendorf T Zillinger T Andryka K Schlee-Guimaraes TM Schmitz S Marx S . Immune sensing of synthetic, bacterial, and protozoan RNA by toll-like receptor 8 requires coordinated processing by RNase T2 and RNase 2. Immunity. (2020) 52:591–605.e6. doi: 10.1016/j.immuni.2020.03.009. PMID: 32294405

[20] Aroca-Crevillén A Vicanolo T Ovadia S Hidalgo A . Neutrophils in physiology and pathology. Annu Rev Pathol. (2024) 19:227–59. doi: 10.1146/annurev-pathmechdis-051222-015009. PMID: 38265879 PMC11060889

[21] Mohammadi N Midiri A Mancuso G Patanè F Venza M Venza I . Neutrophils directly recognize group B streptococci and contribute to interleukin-1β Production during infection. PloS One. (2016) 11:e0160249. doi: 10.1371/journal.pone.0160249. PMID: 27509078 PMC4980021

[22] De Gaetano GV Famà A Lentini G Coppolino F Venza M Venza I . Role of endosomal toll-like receptors in immune sensing of Klebsiella pneumoniae. Front Immunol. (2025) 16:1538425. doi: 10.3389/fimmu.2025.1538425. PMID: 40248691 PMC12003421

[23] Lentini G Famà A De Gaetano GV Galbo R Coppolino F Venza M . Role of endosomal TLRs in staphylococcus aureus infection. J Immunol. (2021) 207:1448–55. doi: 10.4049/jimmunol.2100389. PMID: 34362834

[24] Carey RB Eisenstein TK Shockman GD Greber TF Swenson RM . Soluble group- and type-specific antigens from type III group B Streptococcus. Infect Immun. (1980) 28:195–203. doi: 10.1128/iai.28.1.195-203.1980. PMID: 6155346 PMC550912

[25] Mancuso G Gambuzza M Midiri A Biondo C Papasergi S Akira S . Bacterial recognition by TLR7 in the lysosomes of conventional dendritic cells. Nat Immunol. (2009) 10:587–94. doi: 10.1038/ni.1733. PMID: 19430477

[26] Lentini G De Gaetano GV Famà A Galbo R Coppolino F Mancuso G . Neutrophils discriminate live from dead bacteria by integrating signals initiated by Fprs and TLRs. EMBO J. (2022) 41:e109386. doi: 10.15252/embj.2021109386. PMID: 35112724 PMC8886525

[27] Lentini G Famà A Biondo C Mohammadi N Galbo R Mancuso G . Neutrophils enhance their own influx to sites of bacterial infection via endosomal TLR-dependent cxcl2 production. J Immunol. (2020) 204:660–70. doi: 10.4049/jimmunol.1901039. PMID: 31852751

[28] Zhou C Gao Y Ding P Wu T Ji G . The role of CXCL family members in different diseases. Cell Death Discov. (2023) 9:212. doi: 10.1038/s41420-023-01524-9. PMID: 37393391 PMC10314943

[29] Stenfeldt AL Karlsson J Wennerås C Bylund J Fu H Dahlgren C . Cyclosporin H, Boc-MLF and Boc-FLFLF are antagonists that preferentially inhibit activity triggered through the formyl peptide receptor. Inflammation. (2007) 30:224–9. doi: 10.1007/s10753-007-9040-4. PMID: 17687636

[30] Bae YS Lee HY Jo EJ Kim JI Kang HK Ye RD . Identification of peptides that antagonize formyl peptide receptor-like 1-mediated signaling. J Immunol. (2004) 173:607–14. doi: 10.4049/jimmunol.173.1.607. PMID: 15210823

[31] Fu H Dahlgren C Bylund J . Subinhibitory concentrations of the deformylase inhibitor actinonin increase bacterial release of neutrophil-activating peptides: a new approach to antimicrobial chemotherapy. Antimicrob Agents Chemother. (2003) 47:2545–50. doi: 10.1128/AAC.47.8.2545-2550.2003. PMID: 12878517 PMC166101

[32] Moen SH Ehrnström B Kojen JF Yurchenko M Beckwith KS Afset JE . Human toll-like receptor 8 (TLR8) is an important sensor of pyogenic bacteria, and is attenuated by cell surface TLR signaling. Front Immunol. (2019) 10:1209. doi: 10.3389/fimmu.2019.01209. PMID: 31214180 PMC6554558

[33] Zhang S Hu Z Tanji H Jiang S Das N Li J . Small-molecule inhibition of TLR8 through stabilization of its resting state. Nat Chem Biol. (2018) 14:58–64. doi: 10.1038/nchembio.2518. PMID: 29155428 PMC5726935

[34] Mistry P Laird MH Schwarz RS Greene S Dyson T Snyder GA . Inhibition of TLR2 signaling by small molecule inhibitors targeting a pocket within the TLR2 TIR domain. Proc Natl Acad Sci USA. (2015) 112:5455–60. doi: 10.1073/pnas.1422576112. PMID: 25870276 PMC4418912

[35] Takeda K Kaisho T Akira S . Toll-like receptors. Annu Rev Immunol. (2003) 21:335–76. doi: 10.1146/annurev.immunol.21.120601.141126. PMID: 12524386

[36] Henneke P Dramsi S Mancuso G Chraibi K Pellegrini E Theilacker C . Lipoproteins are critical TLR2 activating toxins in group B streptococcal sepsis. J Immunol. (2008) 180:6149–58. doi: 10.4049/jimmunol.180.9.6149. PMID: 18424736

[37] Ehrnström B Kojen JF Giambelluca M Ryan L Moen SH Hu Z . TLR8 and complement C5 induce cytokine release and thrombin activation in human whole blood challenged with Gram-positive bacteria. J Leukoc Biol. (2020) 107:673–83. doi: 10.1002/JLB.3A0120-114R. PMID: 32083344

[38] Jurk M Heil F Vollmer J Schetter C Krieg AM Wagner H . Human TLR7 or TLR8 independently confer responsiveness to the antiviral compound R-848. Nat Immunol. (2002) 3:499. doi: 10.1038/ni0602-499. PMID: 12032557

[39] Signorino G Mohammadi N Patanè F Buscetta M Venza M Venza I . Role of Toll-like receptor 13 in innate immune recognition of group B streptococci. Infect Immun. (2014) 82:5013–22. doi: 10.1128/IAI.02282-14. PMID: 25225249 PMC4249301

[40] Famà A Midiri A Mancuso G Biondo C Lentini G Galbo R . Nucleic acid-sensing toll-like receptors play a dominant role in innate immune recognition of pneumococci. mBio. (2020) 11(2):e00415-20. doi: 10.1128/mBio.00415-20. PMID: 32209688 PMC7157524

[41] Rodriguez-Rodrigues N Castillo LA Landoni VI Martire-Greco D Milillo MA Barrionuevo P . Prokaryotic RNA associated to bacterial viability induces polymorphonuclear neutrophil activation. Front Cell Infect Microbiol. (2017) 7:306. doi: 10.3389/fcimb.2017.00306. PMID: 28730145 PMC5498479

[42] Jin T Brefo-Mensah E Fan W Zeng W Li Y Zhang Y . Crystal structure of the Streptococcus agalactiae CAMP factor provides insights into its membrane-permeabilizing activity. J Biol Chem. (2018) 293:11867–77. doi: 10.1074/jbc.RA118.002336. PMID: 29884770 PMC6066297

[43] Pietrocola G Schubert A Visai L Torti M Fitzgerald JR Foster TJ . FbsA, a fibrinogen-binding protein from Streptococcus agalactiae, mediates platelet aggregation. Blood. (2005) 105:1052–9. doi: 10.1182/blood-2004-06-2149. PMID: 15383464

