# Supplementary material for: Human neutrophils recognize group B streptococci via formylated peptide receptors and toll-like receptor 8
Source: Front Immunol. 2026 May 4;17:1828994. doi: 10.3389/fimmu.2026.1828994 (PMC13180625; doi:10.3389/fimmu.2026.1828994)
Supplement: Supplementary file 1 [file DataSheet1.pdf]

Supplementary Material

Fig. S1

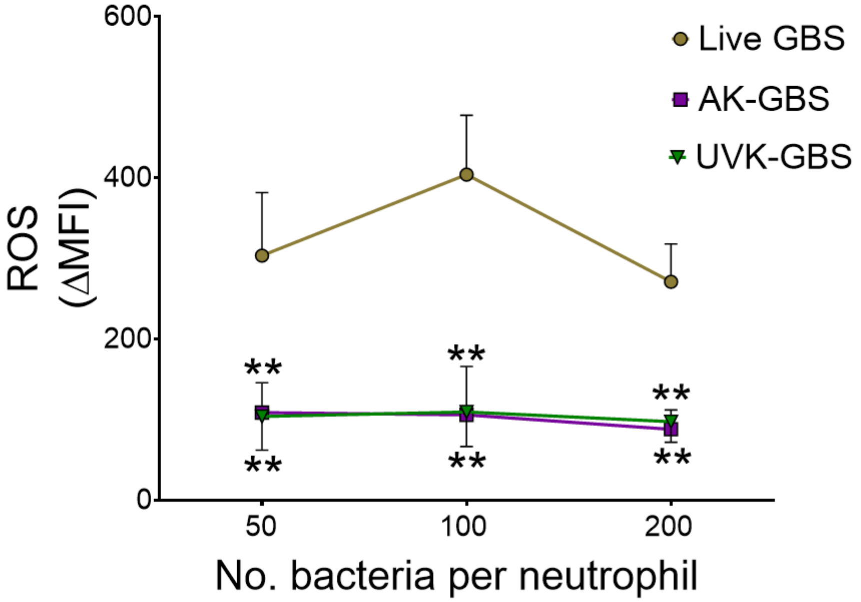

Fig. S1 GBS killed by ultraviolet light or antibiotics are weak inducers of reactive oxygen species. Reactive oxygen species released by neutrophils stimulated with the indicated numbers of GBS per neutrophil. AK-GBS, antibiotic-killed GBS; UVK-GBS, GBS killed by exposure to ultraviolet light. Data are expressed as means ± standard deviations from five independent experiments, each performed in duplicate. **$p < 0.01$, as determined by the Mann-Whitney test; ns, not significant.

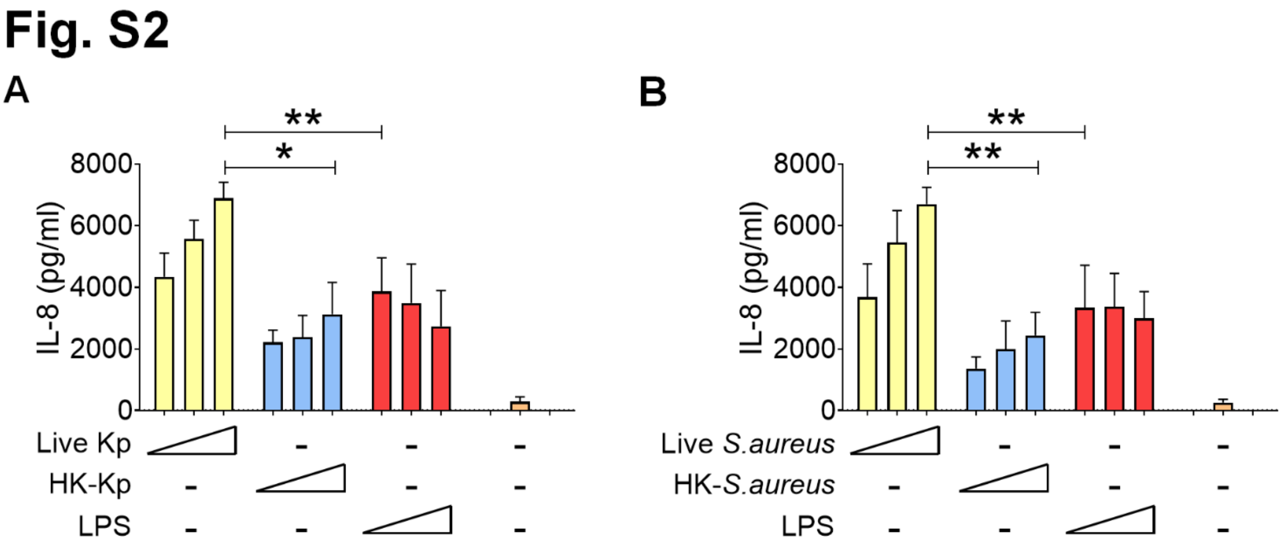

Fig. S2. High-level IL-8 production by live *Klebsiella pneumoniae* and *Staphylococcus aureus.* (**A**) Release of IL-8 following stimulation with live (MOIs of 2, 5, and 10) or heat killed (HK; 10, 25 and 50µg/mL) *Klebsiella pneumoniae* (Kp) (**A**) or *Staphylococcus aureus* (**B**). *Escherichia coli* lipopolysaccharide (LPS; 10, 100 and 1000ng/mL) was included as a positive control. Data are expressed as means ± standard deviations from three independent experiments, each performed in duplicate. **p < 0.01, as determined by the Mann-Whitney test; ns, not significant.

Fig. S3

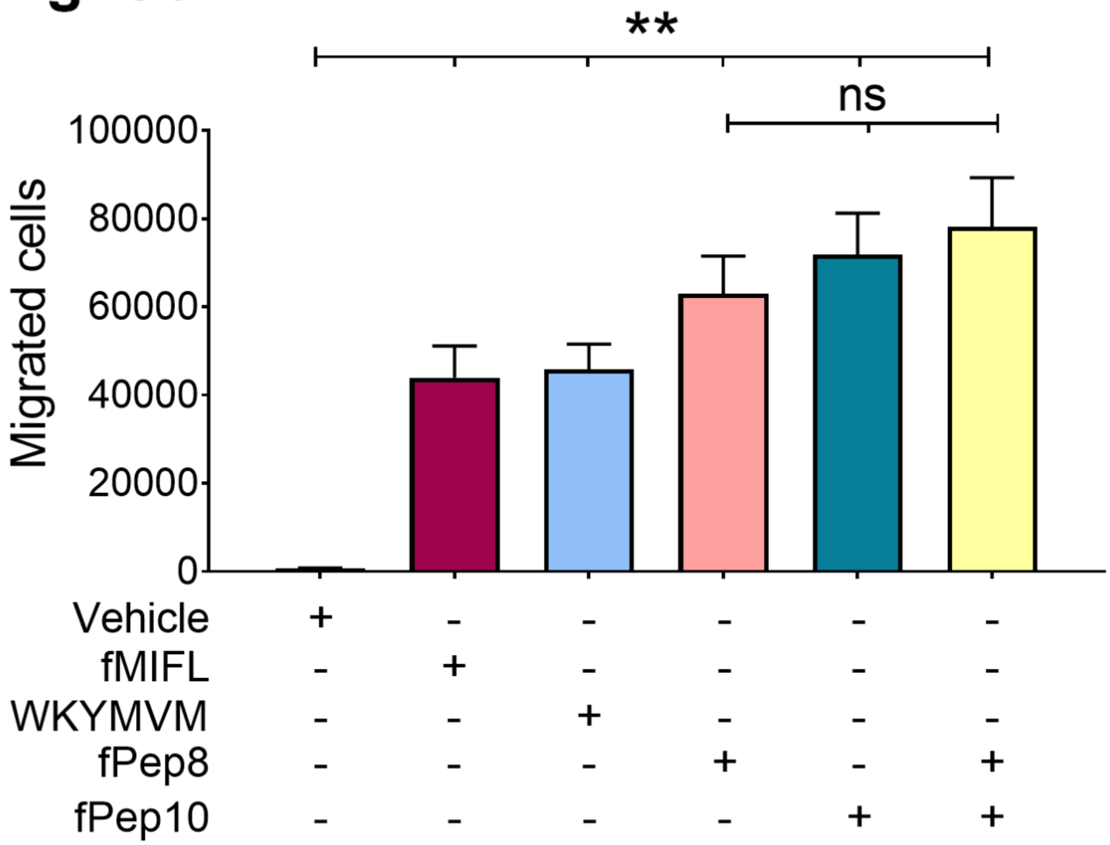

Fig. S3. Chemotactic response to a combination of GBS formylated peptides.
Neutrophils were allowed to migrate towards GBS formylated peptides fPep8 or fPep10 or their combination (1 µM each). The formylated peptides fMIFL and WKYMVM (both at 1µM) were used as positive controls. Vehicle, 0.2% DMSO. Data are expressed as means ± standard deviations from five independent experiments, each performed in duplicate. **$p < 0.01$, as determined by the Mann-Whitney test; ns, not significant.

Fig. S4

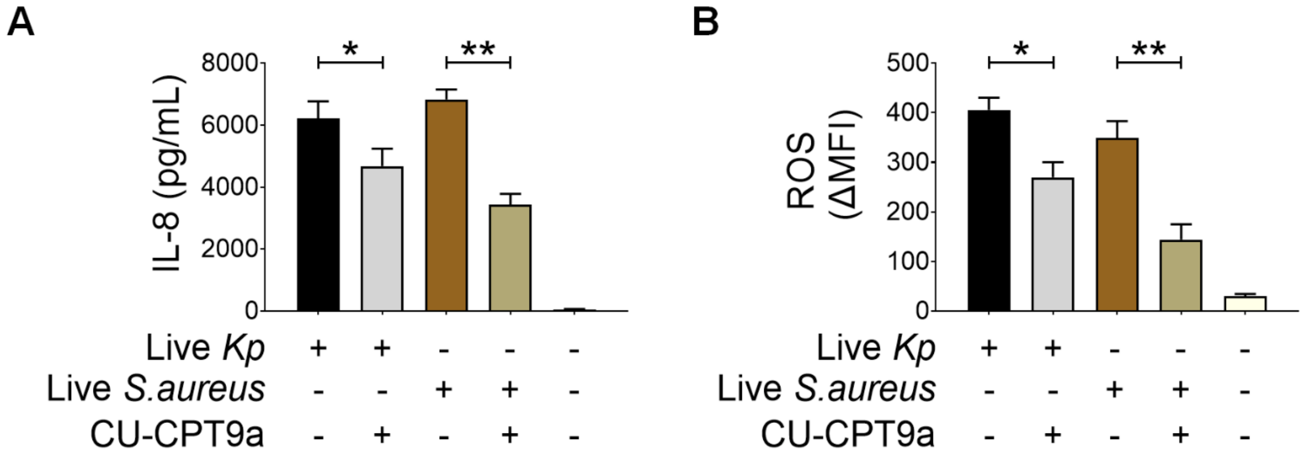

Fig. S4 TLR8 is required for the induction of IL-8 and ROS by *Klebsiella pneumoniae* or *Staphylococcus aureus* in neutrophils.

Effect of pre-treatment with the TLR8 inhibitor CU-CPT9a (3µM) on the release of IL-8 (**A**) and ROS (**B**) after stimulation of neutrophils with live *K. pneumoniae* (*Kp*) or *Staphylococcus aureus* (*S. aureus*, both at a MOI of 100). Data are expressed as means ± standard deviations from three independent experiments, each performed in duplicate. *p < 0.05 and **p < 0.01, as determined by the Mann-Whitney test; ns, not significant.

Fig. S5

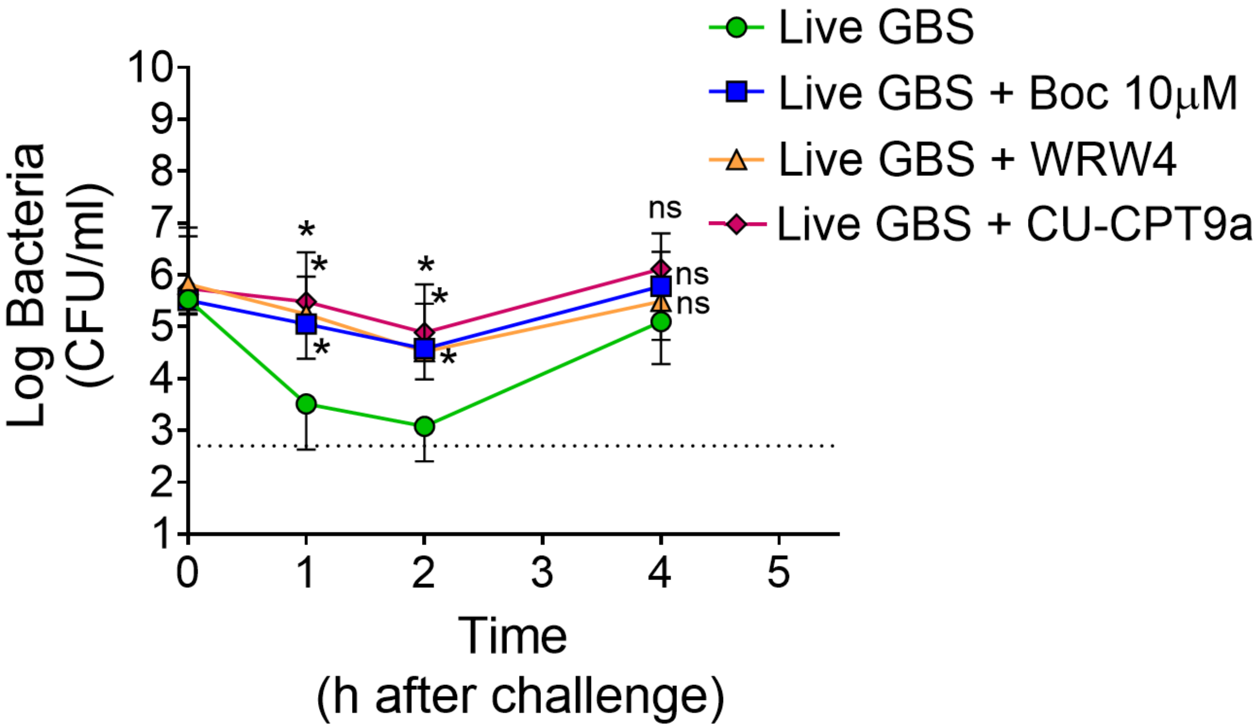

Fig. S5. Engagement of TLR8, FPR1, and/or FPR2 enhances neutrophil killing of live GBS. Neutrophils (5 x 10^5 cells/well) were pre-treated with Boc-2 (10μM), WRW4 (5μM) or CU-CPT9a (3μM) before stimulation with live GBS (4x10^5 CFU/ well). Cells were incubated at 37°C with 5% CO_2 for different times before determination of CFU numbers in cell lysates. Shown are means ± standard deviations from three independent experiments, each performed in duplicate. *$p < 0.05$ as determined by the Mann-Whitney test; ns, not significant.
